# A case study addressing trauma needs during COVID-19 remote learning from an ecological systems theory framework

**DOI:** 10.1186/s40359-022-00848-y

**Published:** 2022-05-31

**Authors:** Sharmeen Mahmud

**Affiliations:** Pepperdine University, Malibu, CA USA

**Keywords:** Online learning, Social emotional learning, Trauma, Teacher student relationships, Pandemic, Teacher perceptions

## Abstract

Mental health conditions related to trauma among American children are a concern, particularly because of the impacts of the COVID-19 pandemic. Children, as students, carry the trauma they encounter with them into the classroom. Students impacted by trauma learn differently due to effects on the brain that relate to several impairments, causing them to perform poorly in school. However, teachers may not always understand this issue. This case study shows how certain dynamics within the EST layers impacted one school during the pandemic. The purpose of this study was to examine how teachers at the school experienced a trauma-informed online PD and SEL program intended to improve student outcomes, teacher perceptions, and teacher–student relationships. The six participants included teachers in a K-8 low-income, minority population charter school. The assessment tools used were the Teacher–Student Relationship Scale, Teacher Perception Scale, and Student Outcomes Survey. The teachers’ outlook on SEL improved, particularly online. This improvement helped the teachers implement community circles and SEL infused with mindfulness in their online classrooms, which may have helped them maintain their relationships with the students and may have helped the students with academic and stress outcomes. During unprecedented times, the maintenance, rather than the deterioration, of student outcomes and teacher–student relationships is an accomplishment and an area that necessitates further research.

## Introduction

Students impacted by trauma may learn differently due to effects on cognitive performance and the brain that cause low executive functioning, poor self-regulation skills, and memory and visual-learning impairments, causing them to perform poorly in school [1–4]. The National Institute of Mental Health [5] defines a child’s trauma experience as “emotionally painful, shocking, stressful, and sometimes life-threatening”. When students experience such changes affecting the students’ ability, teachers may face teaching difficulties [6]. Additionally, trauma-impacted students may behave differently [5–7]. Thus, classroom management becomes an issue in that teachers need to be equipped with trauma-informed practices and social emotional learning (SEL) curricula [6–9]. When teachers misinterpret trauma symptoms as behavioral problems, their response to students is often harmful to the learning process [6, 10, 11]. Student behavioral problems and teachers’ assumptions have led to high teacher turnover rates and unproductive environments in which extremely stressed teachers work with highly stressed students [12–14].

Mental health conditions related to trauma among American children are a concern, particularly because of the impacts of the COVID-19 pandemic, which has led to collective trauma [15, 16]. Collective trauma occurs when a traumatic event is experienced by an entire society, leaves lasting memories impacting future generations [17] and includes world health crises such as COVID-19. Although children can receive trauma treatment through therapy and medical care, they often do not due to a lack of parental awareness and opportunity [18]. Schools can become cornerstones for children’s wellbeing, as children spend much of their time in the school setting [19]. Through schools, mental health becomes much more accessible, and children are more likely to receive the help they need [19]. As educators become the first to reach these children, teachers are placed in the healing process's foreground [19]. However, if teachers are not equipped to understand these children’s trauma-inflicted behaviors, then these students may not get the help that they need and may suffer academically.

The research problem addressed in this study was the negative impact of trauma on student learning outcomes, particularly during the COVID-19 pandemic. Several articles emphasized the need for trauma informed practices and connections in the classroom with the increase of trauma among students, particularly since the COVID-19 pandemic [6, 7, 20, 21]. A study exploring needs of students with past trauma in the classroom, acknowledges that this is becoming the daily undertaking of teachers and identifies required teacher efficacy in the areas of social emotional learning to support these at-risk students [22]. Research indicates that teachers need to engage students in social emotional learning by establishing safe environments and building relationships, which requires time and understanding of students as well as programs [22]. It has also been found that building relationships with students requires a relational perspective of interpersonal communication between teacher and student, rather than self-reflection by teachers, and that this perspective can be the focus of any teacher [23]. Yet, research shows that teachers who prioritize building relationships with their students and teaching them social emotional skills find the work challenging without the availability of trauma-informed trainings [20]. While there is some research addressing the need for bonding activities in the classroom that help build teacher student relationships during COVID-19 [20, 22, 23], they do not specifically address how to do these relationship building activities in online classrooms, such as the use of community circles and mindfulness. This study informs teachers about community circles and mindfulness to build teacher-student relationships and attempted to change teacher perceptions of the possibilities of their use to help alleviate trauma symptoms in the online classroom.

### Underlying factors affecting the underperformance of trauma-impacted students

The factors related to and potential underlying causes for the underperformance of trauma-impacted students were framed in Bronfenbrenner’s [24] ecological systems theory (EST). EST posits that individuals interact within five environmental systems: the microsystem, mesosystem, exosystem, macrosystem, and chronosystem [25]. The five EST layers contain the core of a student’s environment, and this study focuses on the microsystem, exosystem, and chronosystem. In the microsystem, family and teachers influence children. Once children start school, teachers become an important part of the microsystem, as they work daily with children, generally at least five days per week. Among the many significant underlying causes in the microsystem, teachers can be a primary factor in the educational outcome of students who have encountered trauma [26–28]. Studies have consistently found a positive correlation between warm, supportive teachers and trauma-impacted students’ success [26, 29, 30]. Warm relationships, in which there is trust between teachers and students, are positively associated with school adaptation, while teacher–student relationships dominated by conflict are negatively associated with school adaptation [26].

Students may face circumstances entirely out of their control and unrelated to them. This environmental layer in a student’s life is the exosystem. Factors in this layer interact with microsystem factors, apart from the student, to impact the student’s development. For example, school policies and budgeting decisions can impact a teacher’s mindset, which can affect how a student is taught. Milkie and Warner [27] found that the lack of material resources is related to students’ mental health. When teachers feel that they do not have the resources to help students with greater needs, such teachers may give up and be less engaged in helping their students. On the other hand, opposite student outcomes were found for teachers who receive support in helping their students [27]. Teachers who have positive influences in their lives showed a relation to positive student outcomes in a qualitative phenomenological study in which teachers were trained in positive psychology strategies [31]. When teachers had supportive strategies, they reflected on being more calm and able to commit to more one-on-one time with students [31], which could help build teacher–student relationships. Teachers reported more positive outcomes for their students, as they were calmer in the classroom, more engaged, and completed more work [31]. This study showed that teacher training could positively influence teachers, and school administrations should consider professional development (PD) for teacher wellbeing, which may more often than not be overlooked and leave teachers feeling unsupported.

Life-impacting events such as natural disasters, divorce, or even traumatic human-made disaster experiences such as school shootings are considered chronosystem factors. Chronosystem factors change the environment in which a person lives as well as the behaviors or nature of a person, weaving themselves into the other layers of the person’s life [25]. This study's time frame is in 2020, under the impacts of COVID-19. The school in which the study was conducted was under school closure mandates and carrying out distance learning [32, 33]. The majority of the student population in the school live in poverty. These children are expected to be more disadvantaged due to school closures because they relied on the school for meals and their home environments may not have been conducive to learning at home [33]. The COVID-19 school closures and resultant distance-learning circumstances meant that the learning gap among low-income students might be enlarged. COVID-19 was a factor that changed the environment in which students lived, with guidelines for social distancing and health precautions. In addition to the learning disruption that this large societal circumstance may have presented, it also posed a sense of threat and danger, impacting the students socially and emotionally. These life-threatening feelings and thoughts can have lengthy impacts [34]. Children living through tragic societal situations should not be overlooked because the danger is real to them. If educators remained untrained on the after-effects of such large-scale impacts on the chronosystem, student learning is likely to suffer due to misunderstandings and the lack of appropriate support [34].

While all layers of EST define the student, the focus of this study was on the microsystem, exosystem, and chronosystem. These layers of EST were chosen to focus on because research indicated that teacher-student relationships were important in the student learning process, particularly for students who experienced trauma [20, 22, 27, 35, 36]. It was also obvious that there were traumatic impacts that can interfere with student learning during COVID-19 [5, 15]. It appeared to be important to address teacher perceptions about SEL and trauma among students under these circumstances. This study does not directly address the macrosystem and mesosystem because they are not within the scope of the study. However, as the layers of the EST framework are interrelated, the values and cultures within the school which fall in the macrosystem were affected as SEL tools ware implemented in the school and teacher perceptions about these tools evolved. Likewise, the factors in the mesosystem were impacted as well, however, it was outside the scope of this study.

### Addressing Chronosystem-related trauma in the classroom

This study focused on teacher–student relationships in the microsystem and teacher perceptions in the exosystem to address the concerns of traumatic stress from the chronosystem. The study addresses the boundaries of online learning on teacher–student relationships and the traumatic impacts that can interfere with student learning during COVID-19. The objective is to improve student outcomes related to grades and behaviors by improving teacher perceptions and teacher–student relationships through teacher training.

#### Teacher professional development

Several researchers have studied teachers’ perceptions about trauma and their abilities to help trauma-impacted students, which involve educating teachers on the neuroscience of trauma [37–39]. Understanding the neurobiology of youth who have undergone trauma is essential for those individuals providing care and services to this vulnerable, at-risk population [40]. Teachers have more empathy for observed behaviors when they receive trauma awareness PD and understand the reasons behind student behaviors [39]. When teachers respond with empathy, they are more likely to positively react to and work through the situation, which causes students to feel that they are cared for and results in better relationships between teachers and students [39]. Providing teacher PD on trauma awareness and the outcomes of trauma effectively supports teacher–student relationships, which is evident in studies that pioneered trauma-informed school movements [38, 39]. As 18 teachers in Melbourne, Australia in government schools started to understand how students were affected by trauma, they were motivated to adjust to student needs and shift their teaching methods [37]. This finding facilitated a greater understanding between teachers and students, and students felt more secure learning from their teachers as the students felt understood [37].

#### Relationship between mindfulness and trauma in the classroom

The relationship between mindfulness and trauma has become central for many studies because “mindfulness is a protective factor against the development of trauma-related psychopathology” [41]. This finding means that students who may face adverse experiences that can cause emotional wounds or trauma can be protected if they practice mindfulness. As the qualities of mindfulness practice are related to children’s “increased awareness and acceptance of their responses to threatening stimuli after exposure to trauma,” it may decrease the degree of PTSD symptoms when a person is subjected to trauma [41], therefore mindfulness may be an effective source of trauma prevention in schools.

Recently, researchers considered whether mindfulness could reduce psychological trauma among children and adolescents after a hurricane [41]. The results indicated a negative relationship between students who used mindfulness strategies and trauma symptoms that were externalized and internalized [41]. Perceived life threats and internalizing symptoms were also lower among students who indicated more mindfulness attributes [41]. The results of this study contribute to the value of mindfulness in helping students exposed to trauma. The effectiveness of teaching mindfulness to students can also be seen in student academic performance [42]. Eight teachers from the United States and Australia teaching mindfulness in their classrooms indicated that they thought when students were taught mindfulness it not only improved academic outcomes, but simultaneously boosted overall wellness [42]. Mindfulness can help to regulate and reduce anxiety, creating the foundation for students to have better relationships. It is a practical way for youth to self-regulate and build resiliency.

#### Community circles help to reduce trauma in the classroom

Building safe connections to help with mindfulness endeavors may be easier if community circles are incorporated to ensure a safe place for students to share their thoughts and experiences while building character and strong student–teacher relationships [43, 44]. Classroom community circles constitute a practice of sitting in a circle with the classroom community while a teacher facilitates safe and engaging conversations [43]. The practice is expected to improve communication and understanding among classroom members, thus enhancing relationships. Silverman and Mee [45] found that community circles in a middle school classroom helped to reduce conflict and led students to feel that they were in a safe environment. A safe environment is a key part of helping students with trauma to reduce their hypervigilance and improve their ability to focus on relevant tasks. Similar results were found in a semirandom controlled trial, in which children in the experimental condition felt safer in the classroom than did children in the control condition, which is important for traumatized children [44]. Elementary school children can relate better to each other, which helps with communication [44]. A study that examined the use of community circles in high schools found that the interaction creates opportunities for teachers and students to become acquainted with each other and gain a sense of community [46]. The study also found that teachers built more positive connections with students from different backgrounds and groups when the teachers used community circles [46]. Therefore, the use of community circles is promising for building teacher–student relationships and providing a safe environment in which students can express themselves. A school community circle is defined by a safe place to have discussions where all students and teachers can see each other’s faces and students can build their community with mutually agreed upon rules and expectations [43, 44]. Therefore, even though not in person, it is quite feasible to conduct a community circle online as all members in an online classroom can see each other’s faces and the teacher can have set expectations, such as to keep cameras on.

## Current study

The present study hoped to improve teacher perspectives of student outcomes in the classroom through improvements in teacher perceptions and teacher–student relationships using PD on empirically founded online SEL tools. A 10-week intervention was designed based on mindfulness, community circles, and teacher PD research. The research questions that guided this study were as follows: (RQ1) Do teachers’ perceptions about working with trauma-impacted students improve after completing the 10-week intervention? (RQ2) How can we characterize teacher–student relationships after the 10-week intervention? (RQ3) To what extent do student stress and academic performance change after the 10-week intervention? (RQ4) Does teacher satisfaction with classroom outcomes improve after the 10-week intervention?

## Methods

This was a convergent mixed-methods design case study that followed the experience of six educators who participated in the intervention at a low-income and minority population transitional kindergarten-to-eighth-grade charter school in San Bernardino, California. The school offers a dual-language (Spanish) immersion program; therefore, the students, staff and teachers are primarily Latinx. The school was physically closed due to COVID-19 during the study. Because remote learning had been implemented and the school had to quickly adapt to it, the teachers and students were adjusting and experiencing high emotional demands. Teachers were teaching a full daily curriculum online as was expected prior to the pandemic. In addition, participating teachers were also including social emotional learning either in the mornings before starting the lesson plan for the day or during a brief break in instruction. Community circles were used virtually during these times.

### Participants

All 35 teachers at the school were invited to participate in the intervention program via email. A convenience sample of six teachers was achieved. The six participants were all fully credentialed teachers ranging from novice first-year teachers to experienced teachers with more than 10 years of teaching experience. Table 1 shows the basic demographics of the teachers.

**Table 1:** Participant demographics

Demographics	Sample
Grade taught	N = 6
First	1
Third	1
Middle School (6–8)	4
Gender	
Male	2
Female	4
Ethnicity	
Latinx	4
Caucasian	1
African American	1
Years of teaching experience	
1–5	1
5–10	4
≥ 10	1

### Procedure

The study was implemented entirely online in three stages: preintervention, intervention, and postintervention. Data were collected during each intervention stage, including the researcher’s field notes.

#### Preintervention

In the preintervention stage, the teachers were emailed the preassessment questionnaires when they responded to the email and agreed to participate in the study. The consent and preassessment questionnaires were completed by a sample of six participants (N = 6). The teachers had two weeks to complete the preassessments prior to starting the intervention.

#### Intervention

The study implemented a teacher-training program based on trauma-informed practices in the classroom, including mindfulness and community circles, which the researcher created and entitled Calm with Character (2C). All components of the intervention program were delivered virtually by the researcher, a licensed mental health professional with a background in mindfulness and school-based therapy. The 10-week intervention included PD in the first week. The two-hour PD addressed trauma-impacted student needs in the classroom and taught the teachers how to use mindfulness and community circles in the classroom to address these needs. Three weeks after the PD (week four), the teachers received a 30-min modeling session in their online classrooms in which mindfulness and community circles were demonstrated with their students. Three weeks later (week seven), a second 30-min modeling session was conducted in the online classrooms. During each modeling session, the participants completed a modeling session checklist that was emailed to them. The researcher prompted the participants to complete the checklist during the modeling session demonstrations. The researcher also collected field notes during the 10 weeks of intervention. As part of the intervention, the participants also received 10 weekly emails with mindfulness lessons and community circle prompts. The teachers received one email on Mondays and a second email on Thursdays with reminders that included the same content and a teacher wellness tip. The lessons incorporated evidence-based community circle protocols and mindfulness content that included breathing techniques, grounding, gratitude, self-compassion, visualization, and movement. The teachers had flexibility and independence as to when they used the lessons in their classrooms, however most incorporated the lessons into short breaks between their regular lessons plans or at the beginning of the day.

#### Postintervention

At the end of the 10 weeks, postassessments that were identical to the preassessments were administered to the participants. The questionnaires were emailed to the participants to complete.

### Measures

#### Teacher–Student Relationship Scale (TSR)—Teacher Version

Brinkworth et al. [47] Teacher–Student Relationship Scale (TSR)—Teacher Version consists of 12 questions and is considered to have both good and undesirable aspects. The TSR shows strong psychometric properties [47] for validity and reliability. The participants responded on a 5-point Likert-type scale, and the measure assessed the quality of the relationship between the teachers and the students from the teachers’ perspectives. Some examples of questions from the scale include the following: (a) “How caring are students toward you?” (b) “How much do you understand your students' personalities?” and (c) “How often do you say something that offends students?”.

#### Teacher Perception Scale

The Teacher Perception Scale assesses a teacher’s sense of resources, knowledge, and training in educating students impacted by trauma. The measure includes 12 questions adapted from the Teacher SEL Belief Scale [48] and a Survey of Teachers’ Knowledge, Perceptions, and Practices [49]. The teachers responded on a 3-point Likert-type scale for the researcher-developed Teacher Perception of SEL (TP) Survey. Some questions on the Survey asked if the teachers understood how to recognize signs of trauma in students and if they were confident in their abilities to identify students with socioemotional or mental health needs and make referrals.

#### Classroom and Student Outcomes Survey

The Classroom and Student Outcomes Survey assesses long-term student outcomes, including student academic performance and stress, as well as teacher satisfaction with their classroom environments. The Classroom and Student Outcomes Survey was composed of two quantitative questions answered on a 4-point Likert-type scale and four open-ended qualitative questions.

#### Modeling Session Checklist

The Modeling Session Checklist consisted of seven questions on a 3-point Likert-type scale, with options to give open-ended responses. A sample question is the following: “I feel comfortable using community circles and doing mindfulness exercises in my classroom.” This instrument was intended to measure the change in teacher beliefs about their classrooms and the student outcomes and their abilities to implement the tools they had received from 2C in the classroom between various time frames. It assessed changes between the PD and first modeling session and changes between the two modeling sessions. The Modeling Session Checklist assessed the short- and mid-term study outcomes.

### Analysis

As the mixed-method convergent design required, the quantitative and qualitative data were analyzed separately per Creswell and Plano-Clark [50]. The results were compared for interpretations of the findings. The researcher considered how the qualitative and quantitative data were related to each other and resulted in a complete understanding of the study.

#### Quantitative data

The quantitative surveys were reviewed for scale scores. The analysis was conducted using the Social Sciences Statistical Program (SPSS) [50]. Descriptive data were computed to explore “general trends” [50] within the variables. Pre- and postchanges in the quantitative data were examined. The effect size of the intervention for each survey was calculated using Cohen’s D statistical analysis. For the instruments, the author noted no biases. Pre-existing quantitative instruments were used which had established reliability and validity.

#### Qualitative data

The qualitative survey data were analyzed using emergent coding by reading through the responses for emerging themes. Common answers showed a common theme, which was coded as a broad category of themes. The code labels came from words extracted from the responses, which involved in vivo coding per Creswell and Plano-Clark [50]. Qualitative codebooks were developed. Regardless of the low participants, qualitative data saturation was reached by the 6th participant. The researcher’s field notes contributed to the qualitative data and incorporated the researcher’s observations during the 10-week intervention. The field notes were then a priori coded to consider themes, such as teacher patterns, school policy relationships, and pandemic impacts. The codes were categorized to develop seven themes. The primary investigator kept memos for the qualitative portion and no biases were observed. The validity of the qualitative questions was determined by using multiple coders and thick, rich field notes. An external reviewer reviewed qualitative questions for validity before the study was approved.

## Results

A profile is given for each participant using the participants’ responses from the questionnaires and the qualitative data to highlight the unique experiences of each participant. Then, the study results are presented and summarized through the four research questions.

### Teacher One

Teacher One showed an improvement in perception toward the feasibility of using SEL and working with trauma-impacted students. According to the qualitative data, this teacher went from using the 2C SEL program once per week at week four to twice per week at week seven, supporting an improved perception of feasibility and comfort level. Based on the researcher’s field notes, this teacher showed an eagerness to learn and help the students and actively sought out interventions and activities. Teacher One showed an improvement from the pre- to the post-TSR. This finding is supported by the qualitative data, which showed that the teacher felt that at week four, communication was ‘somewhat improving’ with the students, but by week seven, communication with the students had improved ‘a lot’, and the students were self-regulating ‘a lot better’. The teacher felt satisfied with the classroom environment at the end of the 10 weeks, stating, “I am satisfied with their behavior. I think that it is on me to make sure that the class environment is welcoming, and I feel that I have pretty good classroom management.” The teacher believed that the students were self-regulating, and that this ability was contributing to maintaining their stress levels. This teacher felt that the student learning outcomes had not improved but neither had they deteriorated. The teacher’s feelings about the learning outcomes were poignantly expressed: “It feels like we are stuck or frozen in regard to student learning outcomes. I think it will be better when we return to in-person learning.”

### Teacher Two

Teacher Two showed a significant improvement from pre- to post-TP. The teacher indicated in the modeling session checklist that he or she consistently used the 2C SEL once per week in the classroom. The teacher stated that he or she was consistently comfortable using SEL. This teacher invited the researcher to model sessions and to work with the class. The teacher told the researcher that the students “truly need this”. The TSR for Teacher Two showed improvement from the pre- to the postassessment, which aligned with the qualitative data in which the teacher indicated that student communication improved more by the seventh week compared to week four. The teacher stated that he or she was satisfied with the classroom environment and took responsibility for its outcomes: “I am fairly satisfied. I think much of it depends on me”. This statement coincides with the student learning outcomes that the teacher indicated, as the learning outcomes were maintained for these students with pre- to post-assessment scores remaining the same and in the high range.

### Teacher Three

Teachers’ perceptions of SEL feasibility and the ability to work with trauma-impacted students were reduced for Teacher Three. This teacher indicated discomfort with SEL at the beginning of the intervention, and he or she stated that they were not using SEL at week four. By week seven, the teacher stated that he or she had begun using SEL at least once a week, but he or she was still uncomfortable using it. This teacher’s discomfort with SEL coincided with the reduced perception of feasibility and the ability to work with trauma-impacted students. The teacher indicated that he or she was satisfied with the classroom environment and student behavior at week four of the study and indicated less satisfaction at week seven. The inability to easily use SEL may have contributed to this result. The teacher was also ambivalent about the student learning outcomes and stress at the end of the ten weeks.

### Teacher Four

The TSR Scale pre- and postassessments remained the same for Teacher Four. The teacher perceived relatively strong relationships with the students that were maintained. The scale results were supported by the qualitative data in which the teacher indicated improvement in communication with the students from week four to week seven. The teacher also felt that the students could self-regulate, and skills had improved throughout the intervention. While the qualitative data indicated that the teacher periodically used SEL in the classroom and he or she was only somewhat comfortable with its use, the TP Scale showed a decrease from pre- to postassessment. The teacher felt that the student learning outcomes were progressing poorly, yet the students were “amazing”. The teacher seemed happy with the students regardless of the struggles with engagement and productivity. This teacher showed discomfort with SEL use and did not use it regularly; coincidentally, the teacher felt that the student academic outcomes were poor.

### Teacher Five

This teacher indicated that he or she used SEL daily. He or she felt that because the students were younger, they had a greater need to learn coping and behavioral skills. The teacher tried more to manage classroom behavior than to focus on academics. The qualitative data indicated that by week seven, the teacher felt communication with the students improved considerably, and the students self-regulated considerably better and controlled their emotions. While the teacher perceived the relationships with the students as improving, as indicated by the qualitative data, the TSR Scale for Teacher 5 showed a decrease in the teacher–student relationship between the pre- and postassessment. Based on the pre- and post-TP scales, the teacher maintained his or her perception of SEL feasibility in the classroom. This perception was confirmed by the weekly use of the SEL. The teacher expressed that he or she was satisfied with the classroom outcomes, as the teacher stated that the students were “engaged online using online programs and technology”. This teacher indicated that the student grades were average, which may have contributed to the satisfaction in the classroom outcomes. However, the students were still stressed according to the teacher.

### Teacher Six

This teacher consistently used SEL in the classroom twice per week during the 10 weeks. According to the qualitative data, the teacher slowly increased in comfort level when using SEL. Accordingly, the TP score improved from pre- to postassessment. This teacher indicated that he or she did not have knowledge of the term SEL in the preassessment; therefore, the change in understanding of SEL and use of the SEL program was progress for this teacher. Although the teacher’s perception of SEL feasibility and the ability to work with trauma-impacted students improved, the TSR score decreased from pre- to postassessment. This finding may be related to the teacher’s lack of satisfaction with the classroom outcomes, as he or she stated, “it could be better.” The teacher felt that the student-learning outcomes were very slow and that the students were not engaged, which could mean that the teacher felt a lack of communication and connection, resulting in the TSR score.

## Summary

Each teacher showed some positive results. The following section presents the study results synthesized according to the research questions.

## Research question one

**Fig. 1:**
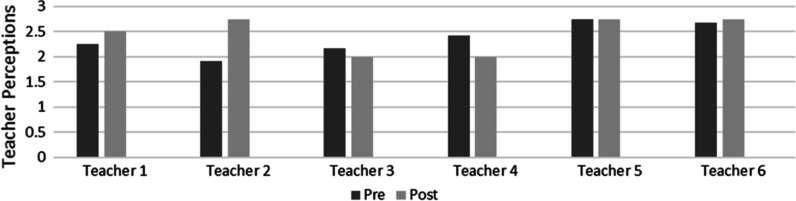
Teachers’ perceptions pre and post intervention

The first research question was do teachers’ perceptions about working with trauma-impacted students improve after completing the 10-week intervention. The pre- and post-TP scale scores were used to measure the change in the teachers’ perceptions about SEL and their abilities to work with trauma-impacted students after working through the study's 2C intervention process. The TP change for each teacher is depicted in Fig. 1. The teachers’ perceptions of SEL and working with trauma-impacted students were also evaluated through the modeling session checklist responses, as the participants indicated their comfort levels and how often they used the 2C SEL with their students.

Three teachers showed improvements in TP, and one maintained. Two teachers showed a reduction in TP. The average TP changed positively between the pre- (M = 2.36, SD = 0.31) and the postscore (M = 2.46, SD = 0.37), with a mean before and after difference of 0.10. The effect size of the intervention was small (0.3) using Cohen’s D statistics. Although the effect size was small, the improvement in teachers’ perceptions about the feasibility of using SEL and working with trauma-impacted students was supported by the qualitative data indicating that most teachers felt that their students were able to self-regulate and that they all increased their use of 2C SEL.

The researcher’s field notes may reveal a reason for the decrease in TP for certain teachers. The student SEL lesson videos received many views, which indicated that the teachers used the videos to help with student wellness. However, in the last three weeks, the lesson video views plummeted, which might be attributed to the increase in the stress the teachers experienced. This finding is because, according to the researcher's field notes, the last three weeks of the study also showed an increase in administration and teacher conflicts. During this time, the school site also experienced an increasing number of COVID-19 cases. Therefore, the teacher focus was not on the SEL emails or lessons at this time. The fact that the postassessments were completed during this critical time may have also impacted the participant responses. The field notes’ themes indicated that the organizational climate contributed to teacher stress and created a threat to the system. Teacher wellness was an important theme that emerged, and it seemed to impact the teachers using the SEL lessons.

## Research question two

**Fig. 2:**
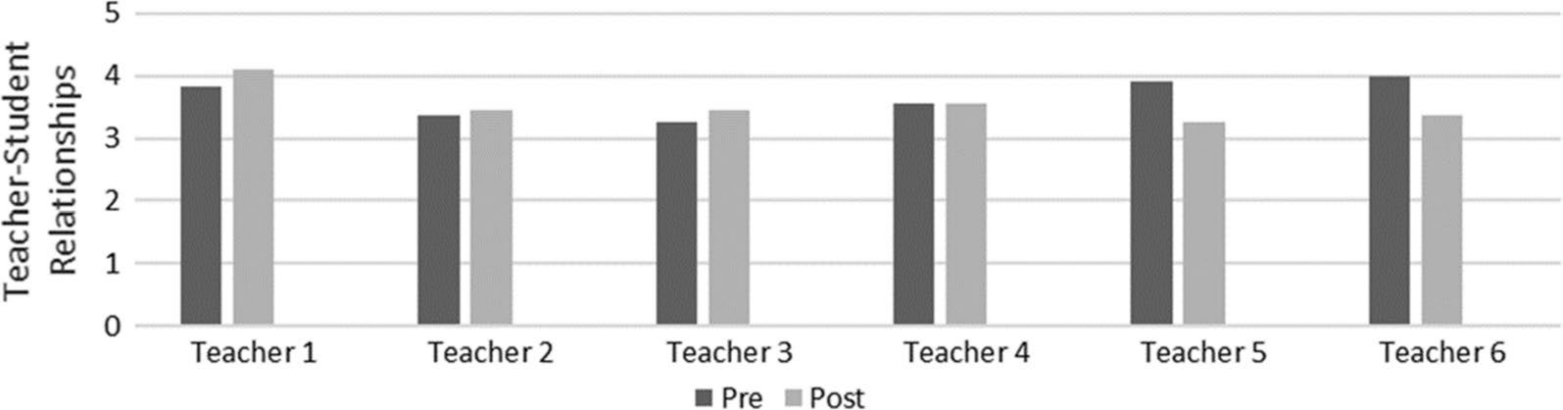
Teacher–student relationships pre and postintervention

The second research question was how can we characterize teacher–student relationships after the 10-week intervention. The change in teacher–student relationships for each teacher is depicted in Fig. 2. Data from the modeling session checklist also supported the results for each teacher’s TSR score. The average score declined between the pre- (M = 3.65, SD = 0.30) and the postscore (M = 3.53, SD = 0.29), with a mean before and after difference of 0.12. The effect size of the intervention was calculated using Cohen’s D statistical analysis. The effect size was found to be small (0.4). Although the average teacher–student relationship slightly declined, it started strong and remained relatively strong with a very small change. Overall, most of the teachers indicated that their communications with the students increased. However, the researcher’s field notes indicated that the teachers struggled to engage the students, which may be connected to the teachers’ abilities to build relationships with their students. The school administration also pressured the teachers to engage the students, as noted in the researcher’s field notes. The teacher–student relationship decrease may also be attributed to teacher stress. Based on the researcher’s field notes, during the entire 10 weeks, a consistent theme was teacher wellness. The teachers showed interest in self-care and appreciated the weekly emails on staff wellness sent to them. Therefore, teacher stress may have been extensive, and they likely needed and sought help with wellness and self-care.

## Research question three

The third research question was to what extent do student stress and academic performance change after the 10-week intervention. This question was assessed using a Student Outcomes Survey (SOS) pre- and postintervention. The SOS had two quantitative and four qualitative questions. The quantitative and qualitative data helped triangulate the findings on the student and classroom outcomes.

The quantitative questions asked about student grades and student stress on a 4-point Likert-type scale. The two questions were as follows:

1. “What are your overall student grades like?”
2. “How stressed are your students?”

Most of the teachers (83.4%) responded that their student grades needed improvement or were average in both the pre- (M = 2.83, SD = 0.41) and the postassessment (M = 2.50, SD = 0.84). The teachers also thought that their students were either somewhat or mostly stressed in both the pre- (*M* = 2.50, *SD* = 0.84) and the postassessment (*M* = 2.50, *SD* = 0.84). Therefore, the quantitative data indicated that the intervention did not improve student grades or stress; however, grades and stress levels were maintained (i.e., they did not deteriorate).

The qualitative data were open-ended survey responses for the student outcomes. The qualitative data triangulated the quantitative data for the student outcome participant responses. The participants responded to four open-ended questions on the student outcomes, and responses were analyzed using emergent coding. The codes were confirmed by three coders. Both the pre- and post-SOS survey results showed one consistent theme: Student learning progress was slow. This finding supported the quantitative results that the teachers thought the student grades were average or needed improvement. The qualitative data indicated that the teachers thought the students were engaged in learning when the teachers provided support and online learning accommodations. The postassessment also indicated that active engagement occurred through teacher-facilitated technology use and collaborative work.

Another theme from the preassessment's emergent coding was that the students learned to self-regulate as a whole class, and the younger students needed extra support. Therefore, the teachers sought additional help for the younger students, which this intervention provided. The analysis of the postassessment indicated that the students could self-regulate despite challenges, which indicated the lack of change in student stress levels in the quantitative data. This finding also indicates that the SEL lessons the teachers used with their classes helped, and the students could manage their stress levels so they did not increase.

## Research question 4

The fourth outcome evaluation research question was does teacher satisfaction with classroom outcomes improve after the 10-week intervention. Teacher satisfaction with classroom outcomes was evaluated through the qualitative data from the SOS, modeling session checklists, and the researcher’s field notes. A theme that emerged from both the pre- and post-SOS qualitative survey questions was that the teachers were satisfied with their classroom environments, considering the pandemic. Although the teachers responded that the student learning progress was slow, the teachers reflected that the students were engaged in the teachers’ efforts and self-regulating. These findings might be the reasons the teachers were satisfied with their classroom outcomes. The teachers were satisfied with behavior and classroom management despite the slow academic progress of their students. The teachers thought the slow progress was due to the pandemic and believed it was acceptable because of uncontrollable circumstances.

However, the researcher’s field notes reflected how the teachers struggled with classroom engagement. The teachers expressed concerns that it was difficult to maintain student engagement due to distance-learning dynamics. Despite this observation, teacher–student relationships were indicated to be relatively strong, according to the post-TSR Scale (*M* = 3.53, *SD* = 0.29). The teachers’ understanding of mindfulness and community circle practices, along with their perceptions of the students’ ability to self-regulate, showed confidence increases, according to Modeling Session Checklists 1 (*M* = 2.57, *SD* = 0.65) and 2 (*M* = 2.89, *SD* = 0.47). These findings might be further reasons the teachers felt satisfied with their classrooms.

## Discussion

This study examined how teachers at a school that is conducting remote learning during COVID-19 experience a trauma-informed online PD and SEL program that could benefit their students. The case study examined teachers’ perspectives on student stress and grades, classroom outcomes, SEL and teacher–student relationships. The findings showed no statistically significant decreases, which was important when considering student grades and behaviors. Considering the pandemic, the students might have become more stressed; however, this finding was not indicated by the surveys. The teachers commented that the students could either maintain their grades or, with additional teacher attention, progress slowly. The teachers also stated that the students self-regulated. Teacher 4 commented, “They [the students] are amazing.” Therefore, 2C might have helped prevent the increase in student stress. Regarding grades, one teacher commented, “It feels like we are stuck or frozen in regard to student learning outcomes.” Although learning progress would be beneficial, the students seemed at least to be holding steady. Overall, the teachers were satisfied with their classroom outcomes because the pandemic created conditions around which they worked. This intervention might have sustained teacher–student relationships and helped the student outcomes remain stable rather than worsen. Success was defined as maintaining stress levels and academic outcomes for the students in the middle of this kind of trauma. The literature indicates that children experiencing trauma display problems in school with learning and behaviors, such as self-regulation [51]; however, this study showed how these concerns could be mediated to prevent escalation.

One valuable outcome was that teacher tolerance for SEL, particularly in an online situation, increased. Although the post-TP Scale did not show a statistically significant change in teachers’ perceptions, the teachers indicated that they became more comfortable using the practices that 2C provided them in an online format. The TP Scale results increased from 33.3% to 83.3% for informal SEL lessons as part of the participants' regular teaching practice. This finding was a significant improvement and demonstrated the achievement of regularly using mindfulness and community circles in the teachers’ classrooms. Studies have shown that even the smallest mindfulness SEL [52, 53] and teacher–student conversations [45] can make vast improvements, notably during traumatic times. Therefore, teacher comfort with SEL tools, such as mindfulness and community circles, may have increased their recognition of the need for teacher–student relationships, which can curb mental health concerns.

The teachers' use of mindfulness and community circles in the classroom showed that the PD, emails, and modeling sessions were effective. The literature indicates that ongoing PD follow up is important, with opportunities for contact and conversations [54, 55]. These opportunities were offered with the two modeling sessions and weekly emails with videos of the lessons. The emails and modeling sessions allowed opportunities for ongoing conversations for the participants, which Mezirow [56] indicated led to critical reflection for growth and transformative learning. State et al. [57] discussed that effective, ongoing training should include reminders about strategies and online modules, which the emails did weekly. State et al. [57] emphasized modeling the concepts taught in PD, which the two modeling sessions did in this study. Research also indicates that for best results the follow up professional development for teachers should incorporate their students [55], which this intervention did through the modeling sessions in the teacher’s classrooms, and this may have helped the results.

It was unexpected that the average teacher-student relationships scores started out fairly strong and remained in the same range. Given the baseline was on the higher end, there was little room for growth. However, in this situation, participant representation should be considered, as Brinkworth et al., [47] indicated, since these participants may have been more interested in working on their teacher-student relationships due to their initiative to volunteer for the study. There may have also been participant response bias as to why teachers stated that their relationships were good from the start and remained good [4, 58]. Research indicates that teachers who have high self-efficacy will indicate stronger teacher-student relationships in comparison to what their students may perceive [59]. The slight decrease in the score, which is not statistically significant could reflect on the impacts of the online learning situation during COVID-19. Literature indicates that there were declines in connections in the classrooms and frustrations in online learning were evident during COVID-19 [13, 60]. As teachers reported more frustrations with their work environment leading to increased stress, this can be a reason behind the reduction in teacher student relationships because higher stress has been found to be related to lower teacher-student relationships [59].

While the increase in stress factors may have impacted the teachers and their interaction with students to a certain level, despite the slight decline the average teacher-student relationship stayed on the positive range of the scale. Supporting teacher wellness might also have helped maintain teacher–student relationships, which the teachers indicated to be strong in both the pre- and posttests; therefore, few changes occurred. Research has indicated that teacher wellness builds positive relationships with children, and teacher-training programs that include mindfulness might contribute to this outcome [61, 62]. When teachers participate in mindfulness training, they build focus, emotional regulation, and tolerance for uncertain times [63]. These mindfulness practices were likely contributors to their wellness and ability to continue focusing on their relationships with students.

This study's process, which included a weekly second email regarding staff wellness and a reminder about the lesson plans, led to some interesting outcomes. Although teacher wellness was beyond this study's scope, the researcher’s field notes regarding these emails showed that they were an important factor. The researcher received random emails and verbal comments from the participants regarding the wellness emails, indicating that the participants appreciated and valued the emails. The high demand for wellness support was not expected in this study. The literature on teacher wellness indicates that teacher wellbeing can change teaching practices in ways that impact student school outcomes [31, 59]. Turner and Theilking [31] found that positive influences, such as positive psychology strategies, related to teachers’ feeling calm and, under such circumstances, teachers could commit to more one-on-one time with students. The wellness strategies that the teachers were emailed each week seemed to have a similar effect.

The researcher's field notes also indicate that teacher wellness was an important part of whether they carried out the weekly SEL lesson plans delivered to them. When the teachers were frustrated or highly stressed due to administrative and COVID-19 dynamics, the video lesson views significantly dropped, indicating that they most likely did not use these platforms. This outcome is congruent with the literature; Milkie and Warner [27] found that school policies and staff relationships can impact a teacher’s mindset, affecting how a student is taught. According to Milkie and Warner [27], teachers were less motivated to help students who required additional support when teachers did not feel supported and connected. Willis and Nagel [28] discussed that empowered teachers could be role models in traumatized children's lives. Although it was not within this study's scope, the literature and current research findings both indicated that maintaining teacher wellness is a significant aspect of student outcomes.

The student stress levels were maintained rather than worsened throughout the study. Therefore, the mindfulness practices taught to them through the 2C program were most likely beneficial. Sheinman et al. [36] demonstrated that mindfulness taught in a whole classroom model helped children cope with daily challenges. Due to the pandemic, the students were challenged daily in this study and needed a source for coping strategies. During the 10 weeks of this study, the teachers offered one such source in the form of mindfulness practices. Simultaneously, the teachers built relationships with the students, in congruence with research that mindfulness helped in this area [64, 65]. Mindfulness programs with routines and space for self-expression ensure safety and security, which helped the students feel comfortable and connected with their teachers, as did the training using the 2C program.

### Limitations and implications for future research

The pandemic that occurred during this study created certain undeniable dynamics. The historical effects of this study could have impacted its results. Historical effects on validity refer to all the events that occurred between the pre- and posttests [66]. According to the researcher’s field notes, many such events were organizationally related to the pandemic, which might have contributed to more stress and complexities. As with any study, this one has limitations. This was a small case study with only six participants. Therefore, this study cannot be assumed relevant to all teachers and students. However, the data are important and require further research on a larger scale. A larger and more diverse sample is needed to make the results of this study generalizable.

## Conclusion

This study showed how certain dynamics within the EST layers impacted one school during the pandemic in an attempt to implement SEL online to improve student outcomes, teachers’ perceptions, and teacher–student relationships. As the study evolved, it became apparent that exosystem influences on the teachers impacted how they used SEL in their classrooms. The teacher wellness component became an important theme in the effective use of SEL. This finding showed the need for schools to begin examining the wellness of their staff to ensure productivity. The chronosystem factors were immense due to the pandemic stressors and impacted all stakeholders at the school, which is even more reason to emphasize staff wellness and SEL.

The current circumstances bring to the forefront the student learning experience, as factors such as trauma and online learning have to be considered. This study reveals some ways of mediating these classroom problems with programs that use trauma-informed training, mindfulness, and community circles. This study also shows how SEL can be conducted virtually in a whole-classroom format. The intervention that was implemented cannot be overlooked. The 2C model was timesaving for the teachers and required no preparation; thus, during hectic circumstances such as a pandemic it could serve as a helpful resource for the teachers. Teachers generally have an overwhelming amount of planning to do, even in the absence of a pandemic, leading many teachers to sideline SEL. The 2C model eliminated the planning and much of the time that SEL lessons could otherwise require. The delivery of the 2C lessons was short, lasting two to eight minutes, with community circles lasting approximately 15 min, depending on the class size. Because teachers need more academic time in classrooms, these short SEL lessons were well received, as each could be used during break times. This finding may be a significant future factor due to interruptions in student learning, particularly as the pandemic requires teachers to learn more about their students’ social situations [33, 67]. The 2C program can easily be led by any interested party; therefore, further research should examine its effectiveness with broader populations and larger sample sizes. Furthermore, 2C should be considered to determine how this type of SEL can be conducted online.

In conclusion, because this study was implemented virtually, it addressed the boundaries of online learning in teacher–student relationships and the unintended traumatic impacts on student learning. Improving interactions and communication between teachers and students will hopefully help teachers to understand their students and student needs better. The teachers were encouraged to build communication strategies and relationships with the students using community circles which may be hampered during online learning. Teachers’ perceptions about SEL and their ability to help trauma-impacted students were targeted through training and follow up. The research found improvements in the teachers’ outlook on SEL, particularly online. This improvement helped the teachers to implement SEL infused with mindfulness and community circles in their online classrooms, which may have helped them to maintain their relationships with students and may have improved students’ academic and stress outcomes. During unprecedented times, the maintenance, rather than the deterioration, of student outcomes and teacher student relationships is an accomplishment and an area that necessitates further research.

## Data Availability

The datasets generated and/or analyzed during the current study are not publicly available due to the nature of this research and participants did not agree for their data to be shared publicly, but are available from the corresponding author on reasonable request.
